# High-speed adaptive optics line scan confocal retinal imaging for human eye

**DOI:** 10.1371/journal.pone.0169358

**Published:** 2017-03-03

**Authors:** Jing Lu, Boyu Gu, Xiaolin Wang, Yuhua Zhang

**Affiliations:** Department of Ophthalmology, School of Medicine, University of Alabama at Birmingham, Birmingham, Alabama, United States of America; Simon Fraser University, CANADA

## Abstract

**Purpose:**

Continuous and rapid eye movement causes significant intraframe distortion in adaptive optics high resolution retinal imaging. To minimize this artifact, we developed a high speed adaptive optics line scan confocal retinal imaging system.

**Methods:**

A high speed line camera was employed to acquire retinal image and custom adaptive optics was developed to compensate the wave aberration of the human eye’s optics. The spatial resolution and signal to noise ratio were assessed in model eye and in living human eye. The improvement of imaging fidelity was estimated by reduction of intra-frame distortion of retinal images acquired in the living human eyes with frame rates at 30 frames/second (FPS), 100 FPS, and 200 FPS.

**Results:**

The device produced retinal image with cellular level resolution at 200 FPS with a digitization of 512×512 pixels/frame in the living human eye. Cone photoreceptors in the central fovea and rod photoreceptors near the fovea were resolved in three human subjects in normal chorioretinal health. Compared with retinal images acquired at 30 FPS, the intra-frame distortion in images taken at 200 FPS was reduced by 50.9% to 79.7%.

**Conclusions:**

We demonstrated the feasibility of acquiring high resolution retinal images in the living human eye at a speed that minimizes retinal motion artifact. This device may facilitate research involving subjects with nystagmus or unsteady fixation due to central vision loss.

## Introduction

Adaptive optics scanning laser ophthalmoscopy (AOSLO) [1] has emerged as an important imaging modality for evaluating retinal structure and function at the cellular and sub-cellular level in the living human eye and living animal eye [2–4]. Over the past decade, AOSLO has expanded from a confocal imaging modality in early years to a regime now including both confocal [1, 5–8] and non-confocal imaging systems [9–11]. These systems produced fine retinal structure such as cone and rod photoreceptors [2, 4, 12, 13], retinal pigment epithelial cells [14, 15], the finest retinal vasculature [16–20], and optic nerve fiber [21] in human subjects with normal chorioretinal health and in patients with retinal diseases. Microscale biometrics derived from AOSLO images have been used for quantitative assessment of retinal health [22] and response to novel treatment [23]. Recent advance in non-confocal imaging empowers AOSLO to study degenerating photoreceptors [10], transparent retinal neurons [24], two-photon process in photoreceptors [11], retinal vasculature [9], and retinal capillary erythrocytes flux and velocity [25] in the living eye.

While the application of AOSLO is burgeoning in basic vision science and clinical research, this imaging modality has been challenged by continuous and rapid eye movement, even when fixating [26–28]. As a scanning imaging system, AOSLO delivers light from a point source (usually formed by the tip of a single mode fiber) onto the retina and collects the back-scattered light from the illuminated retina through either a confocal or a non-confocal mechanism. A complete image is constructed by raster scanning the light point on the retina. Thus, continual retinal motion will cause errors both within a frame (intra-frame) and in successive frames (inter-frame), if the image acquisition speed is not sufficiently faster than eye movement or the imaging systems is not facilitated by real-time eye motion tracking mechanism [29, 30].

Retinal motion affecting imaging may be classified into 3 modes: tremors, drifts, and microsaccades [27, 28]. Tremors are periodic motions with frequency from 40 to100 Hz and amplitude less than 1 arcminute (~5 μm) [31, 32]. Drifts are slow self-avoiding random walks with speed below 30 arcminutes/s (~150 μm/s) and amplitude of 1–8 arcminutes (5–40 μm) between saccades [27, 33]. Microsaccades are jerk-like movement with duration of ~ 25 ms (~40 Hz) and amplitude < 30 arcminutes (~150 μm) [34]. The frame rate of current AOSLO that works with a ‘flying spot’ mechanism (employing a fast scanner and a slow scanner to generate the scanning raster) is typically ~30 frames per second (FPS) for imaging with a digitization of 512 × 512 pixels/frame. Although the imaging frame rate can reach up to 60 Hz by collecting back-scattered light during flying-back of the horizontal scanner [17], the image acquisition speed is still slow compared to eye motion. This impact is especially pronounced in AOSLO because the image is acquired within a small field of view (typically 1°–2° inside the human eye) with high magnification. In a recent study, Cooper et al identified substantial variation in the repeatability of AOSLO measured cell-to-cell spacing, cell density, percentage of 6-sided Voronoi cells, and Voronoi cell area regularity (VCAR), ranging from 4.6% to 13.2% [22]. In particular, the AOSLO derived VCAR was significantly different from that measured from a flood-illumination AO-fundus camera whose images were thought free of intra-frame motion artifact. This study indicates that AOSLO imaging is impaired by intra-frame distortion and emphasizes the need for distortionless imaging.

To mitigate the errors induced by eye motion, software has been developed for image registration [35], and sophisticated hardware based retinal trackers or imaging stabilizers have been designed [6, 29, 30, 36, 37]. Hammer and Burns et al reported a retinal stabilization system with an accuracy of 1.2 arcminutes [6, 36]. Sheehy et al achieved a tracking accuracy of 0.2 arcminute [37, 38]. Yang et al [31] and Zhang et al [32] further improved the tracking accuracy to 0.05–0.07 arcminute with a highly sophisticated tracking system involving wide-field SLO coarse tracking and AOSLO fine stabilization. While these methods were demonstrated with the ability to track eye movement, they increased overall system complexity and costs. Another approach is to increase the image acquisition speed. This may be achieved by line scan imaging [39–41]. Hammer et al reported a non-AO line scan ophthalmoscope whose frame rate was up to 30 FPS [41]. Mujat et al presented an AO line scanning ophthalmoscope with a frame rate of 15 Hz [42]. Line scan imaging has the potential to increase the frame rate by saving the time for generating the light line.

The fact that eye movement frequency can be up to 100 Hz [43, 44] dictates that the ideal imaging acquisition speed should be at least 200 Hz, according to the Nyquist theorem. This paper presents an AO retinal imaging system meeting this requirement and reports reduction of intra-frame motion artifact of the retinal images by high speed line scan imaging.

## Materials and methods

### High speed confocal retinal imaging system

**Fig 1:**
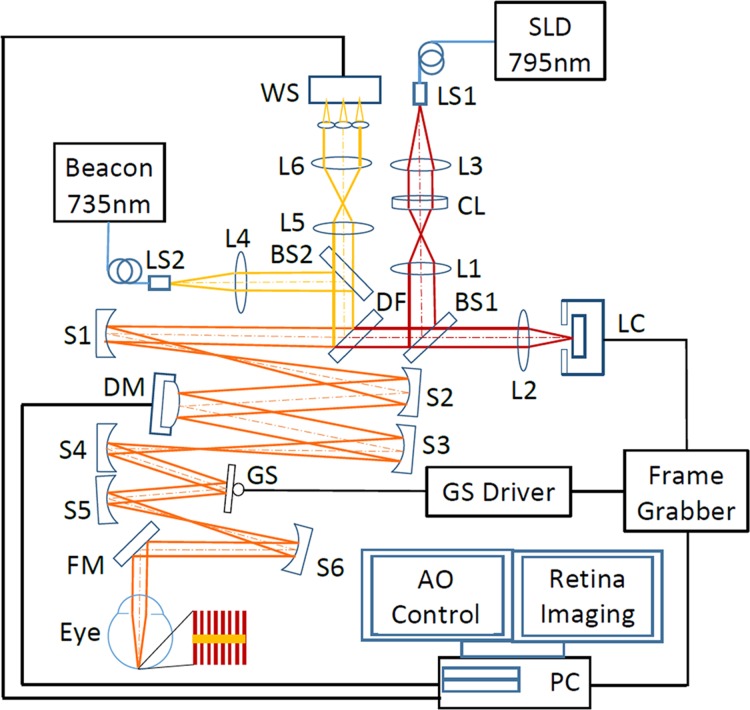
High speed adaptive optics line-scan confocal retinal imaging system. LS1, LS2: Light sources. SLD: Superluminescent diode. CL: Cylindrical lens. DF: Dichroic filter. L1-L5: Lenses. BS1, BS2: Beam splitter. S1-S5: Spherical mirrors. GS: Galvanometric scanner. FM1, FM2: Flat mirrors. WS: Wavefront sensor. DM: Deformable mirror. The imaging path and the wavefront sensing path are indicted by dark red and yellow lines, respectively.

Fig 1 shows the schematic of the high speed AO line scanning retinal imaging system, consisting of three major modules, scanning or retinal imaging optical system, AO system, and retinal image acquisition.

#### Scanning/retinal imaging optical system

The retinal imaging system employs a low coherent superluminescent diode (SLD) (Broadlighter S795-HP, Superlum Ltd., Russia) with a central wavelength of 795 nm and a spectral bandwidth of 15 nm. Light emitting from the single mode fiber of the SLD is collimated and focused by a cylindrical lens (CL) (ACY254-150-B, Thorlabs Inc., NJ) to form a vertical line, which is relayed by an achromatic lens (L1) to beam splitter BS1, which reflects 20% of the light into the scanning optics. Due to the Gaussian profile of the light intensity from the single fiber, the light intensity along the focus line is not uniform. To ensure a uniform illumination, an aperture (CP20S, Thorlabs Inc., NJ) was placed next to the CL, allowing only the central 60% of the beam to be used for imaging. The imaging light is delivered to the eye via the deformable mirror (DM) and the galvanometric scanner (GS) (6200H, Cambridge Technology, Inc., Bedford, MA), the relay optics formed by spherical mirrors S1-S6, forming a raster (red lines next to the eye in Fig 1) scanning on the retina. The diffusely reflected light from the retina transmits inversely along the ingoing path to BS1, through which 80% of the light transmits to imaging lens L2 and is focused on the image sensor of a high speed line camera (spL2048-140km, Basler Co., Germany).

The optical system was designed to meet three major technical conditions. First, the optical resolution, described by the radius of the Airy disc formed by the human eye when it is free of optical aberration, should resolve the cone photoreceptors in the foveal center and the rod photoreceptors, whose size is about 2–3 μm [45]. Second, the digitization of the image should be sufficient for rendering the optical resolution. Specifically, the radius of the Airy disc formed by the human eye should be imaged by at least 2 pixels on the camera [46]. Third, the imaging system should have good confocality to reject out-of-focus scattering and provide fine depth discrimination in the retina. Given a pupil diameter of 6.75 mm, the Airy disk radius on the retina is 2.40 μm. The magnification ratio from the retina to the camera is 14.65 thus the radius of the Airy disc on the camera is 35.16 μm. The pixel size of the line camera is 10 μm × 10 μm, thus the image of the Airy disc is sampled by 3.59 pixels. The field of view (FOV) inside the eye is 1.2° × 1.2°, which is digitized by 512 × 512 pixels. The camera works with the dual-line vertical pixel binning mode. The line imaging chip also serves as an intrinsic line confocal gate whose width is 20 μm, which is 0.28 of the width of the light line focused by collection lens L2, ensuring a highly confocal imaging mechanism [39].

#### AO system

The optical wave aberration of the human eye is measured by a custom Shack-Hartmann wavefront sensor and corrected by a high speed DM (Hi-Speed DM97-15, ALPAO SAS, France). The wavefront sensor consists of a complementary metal-oxide semiconductor (CMOS) camera (MV1-D1312I-160-CL-12, PhotonFocus AG, Switzerland) and a lenslet array (0300–7.6-S, Adaptive Optics Associates, Cambridge, MA). The CMOS camera has enhanced quantum efficiency up to 50% in the infrared region. The lenslets are of a square shape of 0.3 mm × 0.3 mm and a focal length of 7.6 mm. The DM has 97 actuators with stroke up to 30 μm. Wavefront detection employs a beacon with a wavelength of 735 nm (Δλ = 6 nm) from a supercontinuum light source (SuperK Extreme 100 MHz VIS, NKT Photonics AS, Denmark) through an acousto-optic tunable filter (SuperK SELECT, NKT Photonics A/S, Denmark). The beacon is collimated by lens L4 and picked by a beam splitter BS2 with a 5% reflectance to a dichroic filter (T770lpxr, Chroma, Bellows Falls, VT), by which the beacon is fed into the scanning optics and then is relayed to the eye along the same path of the imaging light, forming a scanning line (yellow line in the red scanning raster next to the eye in Fig 1) orthogonal to the lines of imaging light on the retina. The reflected beacon light transmits inversely along the ingoing path to the dichroic filter, where it is directed into the wavefront sensing arm. At BS2, 95% of beacon light transmits through and is relayed by a pair of telescope to the wavefront sensor. The DM, the lenslet array, the Galvanometric scanner, and the collection lens L2 are aligned so that they are all conjugate to the entrance pupil of the eye. The wavefront is corrected for both the ingoing and the outgoing paths.

The control algorithms developed in previous studies [47–49] was adopted for the AO system. The closed-loop frequency of the AO can be up to 50 Hz which is limited by the maximum frame rate of the camera. Retinal imaging and wavefront sensing use the same pupil size. The 1.2° × 1.2° FOV is smaller than the size of the human eye’s wavefront isoplanatic zone [50] thus the wave aberration can be compensated over the whole FOV.

#### Retinal image acquisition

**Fig 2:**
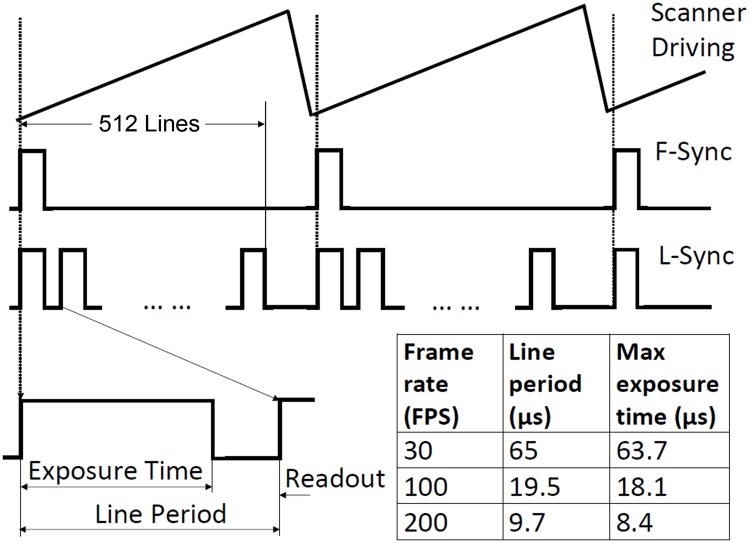
Schematic of the image acquisition timing diagram. The galvanometric scanner is driven by the sawtooth signal generated by a function generator. One period scanning of the galvanometric scanner forms a frame including 512 lines. Each line includes a time for exposure to collect imaging photons (the line exposure time) and a time for reading out the image data (readout time). The line control signal (L-Sync) has two programmable parameters: “Line Period” and “Line Exposure Time.” Given a fixed line numbers per frame, the line period is determined by the frame rate or the time for acquiring a frame, e.g., for a frame consisting of 512 lines, the line period for the frame rate 200 Hz is 9.7 μs, within which a maximum time of 8.4 μs can be used for camera exposure to collect imaging photons, and a minimum 1.3 μs must be reserved for data readout. The table listed the line period time and the maximum exposure time during each line under different frame rates. The L-Sync is sent to the frame grabber. When the frame grabber receives the frame synchronization signal (F-Sync) from the function generator, it starts to record a frame and a counter triggered by the L-Sync will record the number of lines acquired. When the counter reaches 512, the frame grabber will finish acquisition and wait for the next F-Sync. The F-Sync was purposely set with a delay to avoid image distortion at the beginning of each frame.

A frame contains 512 line-images taken by the line camera when the imaging light line sweeps across the FOV. A frame grabber (Radient eCL, Matrox Imaging Co., Canada) acquires the image through CameraLink [51]. A signal generator provides a sawtooth wave to drive the galvanometric scanner. The frequency and amplitude of the sawtooth signal can be adjusted to control the frame rate and the scanning field size. The function generator also provides a synchronization signal of the sawtooth wave (F-Sync), which is used to synchronize the frame grabber and the galvanometric scanner during image acquisition, as illustrated in Fig 2. The line camera generates its own internal line control signal (L-Sync) with two programmable parameters: “Line Period” and “Line Exposure Time.” The L-Sync is also sent to the frame grabber. When the frame grabber receives the F-Sync from the function generator, it will start to record a frame and a counter that is triggered by the L-Sync will record the number of lines acquired. When the counter reaches 512, the frame grabber will finish acquisition and wait for the next F-Sync. To avoid image distortion due to slow scanning speed at the beginning of each frame, the F-Sync was set with a delay (typically 4 line-periods). During the line period of the camera, the line exposure time can be adjusted. With a digitization rate of 512 × 512 pixels/frame, the highest frame rate can be up to 273 FPS. To ensure a good signal to noise ratio (SNR), retinal images were typically acquired at 200 FPS, allowing for a sufficient line exposure time.

### Imaging protocol and processing

The study involved human participants. It followed the tenets of the Declaration of Helsinki and was approved by the Institutional Review Board for Human Use (IRB) at the University of Alabama at Birmingham. Written informed consent was obtained from the participants after the nature and possible consequences of the study were explained.

The power of imaging light (λ = 795 nm) is 0.88 mW, which is 0.4 times of the ANSI max permissible exposures (MPE) under the condition of 1 hour continuous exposure [52, 53]. The power of the wavefront sensing beacon (λ = 735) is 25 μW, ~ 1/10 of the ANSI MPE under 1 hour continuous exposure. The composite MPE for multiple laser exposure (∑ϕ_λ_/MPE_λ_) is 0.5 [52], below the ANSI safety threshold.

To evaluate the benefit of the high speed imaging, retinal images were acquired with different frames rates in human subjects with normal chorioretinal health. The images were registered and averaged to improve the imaging SNR using a custom software [35]. Individual images taken at different retinal locations were manually montaged using image processing software (Photoshop, Adobe System Inc., Mountain View, CA) with a cone-for-cone precision in the overlapped areas.

### Estimate the imaging signal to noise ratio

**Fig 3:**
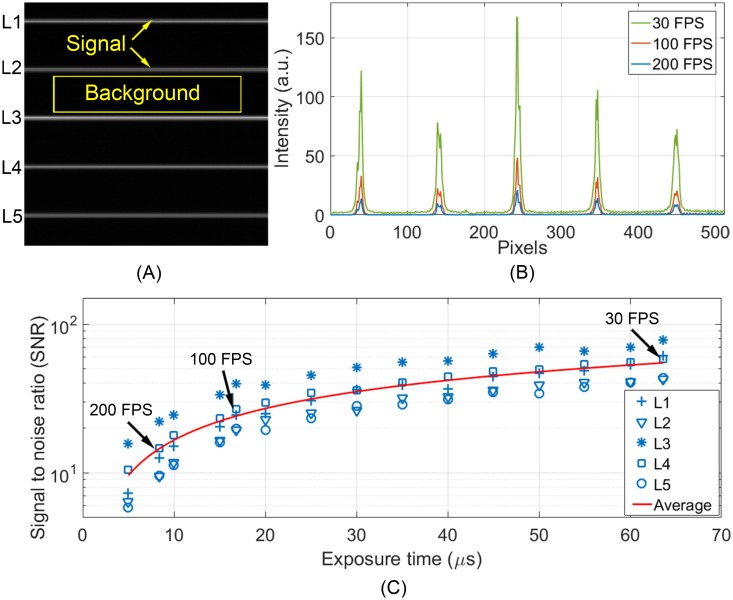
Imaging SNR measured under different frame rates. (A) The testing image for assessing SNR. (B) Intensity profile of 5 bright lines acquired under different frame rates. (C) The SNRs measured under different line exposure time corresponding to different frame rates, as indicated by 5 symbols. Red line is the average SNR of 5 measurements. The mean SNR at 200 FPS, 100 FPS, and 30 FPS are indicated by arrows.

The SNR was estimated using a testing image [54, 55] illustrated in Fig 3A, which included 5 bright lines and the rest of the area was dark (without illumination). The testing image was acquired by placing a diffuse target at the retinal conjugate plane between spherical mirrors S1 and S2. A mask containing 5 small apertures (10.8 μm in diameter) was placed on the focal plane of the cylindrical lens CL and modulated the illuminating line into 5 light spots. The light power of each light spot was approximate 2 μW. Because the target was placed before galvanometric scanner (GS), the image was formed by 5 stationary light spots on the diffuse target with an appearance of 5 uniform bright lines on the dark black background. The intensity along these bright lines (1 × 512 pixels) was considered as the imaging signal, whereas the background noise was measured from the dark area between the bright lines. The SNR was estimated with the equation [55, 56],

$$SNR_{image}=\frac{Mean_{s}-Mean_{b}}{StdDev_{s}}$$ (1)

where *Mean*_*s*_ is the mean of the signal, *Mean*_*b*_ is the mean of the background, and *StdDev*_*s*_ is the stand deviation of the signal. Because of the variation of reflectance on the diffusing target, SNRs assessed from different lines varied accordingly. The overall image SNR was estimated by the average value calculated from 5 lines. The SNR was assessed with images acquired with different frame rates at 30–200 Hz. During the measurement, the camera was set with the same gain and same black level under different frame rates.

### Assess the benefit of high speed confocal imaging

To assess the improvement on reducing intra-frame distortion, we acquired images at the same retinal location (1.5° eccentricity) with frame rate of 30 FPS, 100 FPS, and 200 FPS. During image acquisition, the subject was asked to look at a stationary fixation target and blink naturally.

The method introduced by Vogel et al [35] was used to calculate retinal motion. Briefly, a frame with the least visible distortion was selected from at least 4 candidate frames and used as the reference. Each frame of the video including the reference was divided into 16 stripes (32 lines/stripe). Because a stripe was acquired within a very short time (0.0021 second for a frame consisting of 512 lines acquired at 30 FPS), motion artifact in the stripe was minimal. Instantaneous retinal movements along X and Y direction were estimated by calculating the displacements of stripes in successive frames to the corresponding reference stripe through cross-correlation algorithm. We used the root-mean-square (RMS) of 16 stripes’ displacements along X (horizontal) and Y (vertical) direction in the same frame to estimate the distortion of this frame.

## Results

### Imaging SNR

Fig 3B shows the intensity profiles of 5 light lines imaged at 30 FPS (line exposure time: 63.6 μs), 100 FPS (line exposure time: 16.8 μs), and 200 FPS (line exposure time: 8.4 μs). Fig 3C plots the SNRs of the 5 lines under different exposure time (5 symbols) and the mean SNR (red line). The mean SNR for 30 FPS, 100 FPS, and 200 FPS are 54.93, 24.1, and 14.56, respectively.

### Photoreceptor imaging

**Fig 4:**
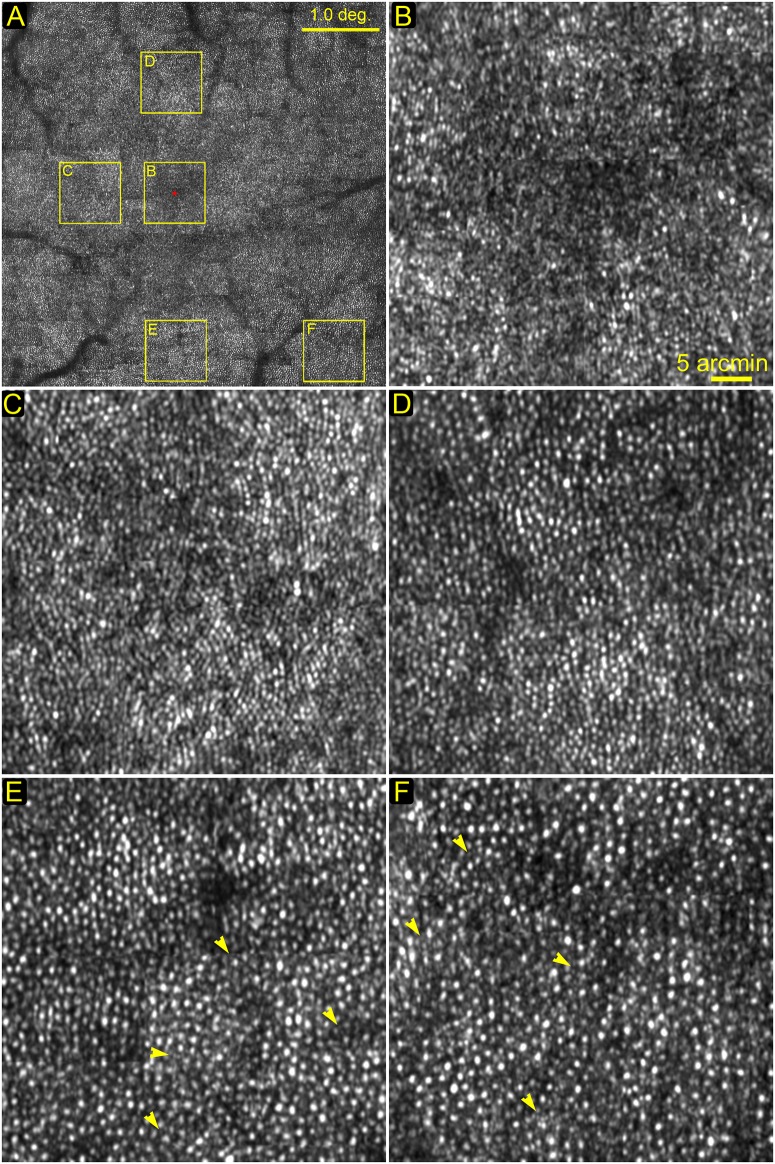
Photoreceptors imaged at different locations. (A) The montage of the central macula, yellow boxes indicate retina areas shown in the following panels. (B) The foveal center. All cells are cone photoreceptors. Except for the very center area (< 0.2°), most cones were resolved. (C) 1.0° eccentricity temporal to the foveal center. All cells are cone photoreceptors. (D) 1.5° eccentricity superior to the foveal center. (E) 2.0° eccentricity inferior to the foveal center. The dark band is a blood vessel shadow. Yellow arrowheads point to rods. (F) 3° eccentricity nasal-inferior to the foveal center. Rods are smaller dots (indicated by yellow arrowheads) surrounding cones which are bigger and brighter. All images were acquired at 200 FPS, and then registered and averaged a set of 50 successive frames using custom software [35]. The scale bar in panel (B) also applies to (C)—(F).

Fig 4A shows a montage of the central macula of a human subject in normal chorioretinal health. All individual images were acquired at 200 FPS. Photoreceptor (mostly are cones) images at 0°, 1.0°, 1.5°, 2°, and 3°eccentricities from the foveal center are shown in Fig 4B–4F. Foveal cone photoreceptors were resolved except for the very foveal center area (< 0.2°) (Fig 4B). In this system, the camera was vertically oriented, acting as a confocal aperture in the horizontal direction while allowing light along the camera dimension (vertically) to superimpose (convolution). Thus, this configuration resulted in slightly poorer image contrast along vertical direction (Fig 4C) [39]. Rod photoreceptors are visible in images taken near the fovea, as shown in Fig 4E (2° inferior) and Fig 4F (3° nasal-inferior) by yellow arrowheads. Although rods may be better visualized in peripheral macula due to high density and large size, here we show rods near the rod free zone [45] with the purpose to demonstrate the lateral resolution. The nearest place where rods are resolved to the foveal center is at approximately 2° eccentricity in our image, as shown in Fig 4E.

### Retinal sectioning

**Fig 5:**
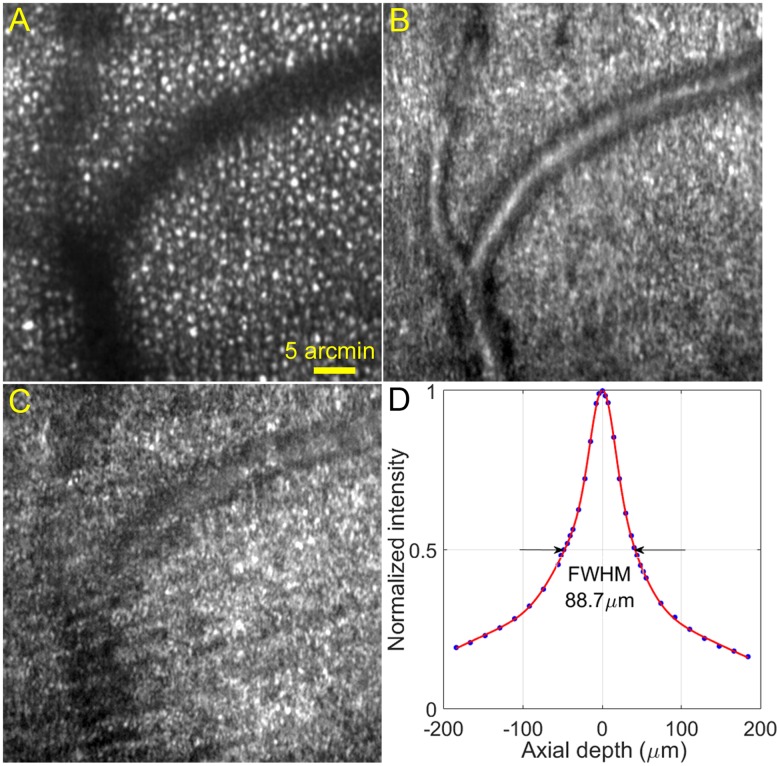
Retinal images acquired at different layers and system axial resolution. The images were taken at approximately 3° eccentricity from the foveal center nasally. (A) The imaging light focused on the photoreceptor layer. The dark bands are shadows of retinal blood vessels. (B) The imaging light focused on the retinal blood vessels between the photoreceptor layer and the optic nerve fiber layer. (C) The focal plane is at the surface of the optic nerve fibers. (D) System axial resolution. All images were acquired at 200 FPS, and then registered and averaged a set of 50 successive frames using custom software [35]. The scale bar in (A) also applies to (B) and (C).

A major technical merit of confocal imaging is the ability to optically ‘section’ the retina, as has been demonstrated since the first AOSLO [1, 57]. With the method introduced by Romero-Borja et al [57], we calibrated the depth discrimination ability of our high speed imaging system. Briefly, a model eye consisting of an achromatic lens (mimicking the cornea and the lens of the human eye’s optics) and a diffusing target (serving as the retina) was placed at the eye’s position. The model retina was moved axially with a micrometer. The average intensity of the image was recorded and plotted against the axial distance that the model retina was moved from the best focus. The full width at half maximum (FWHM) of the intensity-distance plot was considered as the axial resolution of the imaging system with the model eye. After converting to human eye’s coordinate, the axial resolution was 88.7 μm (Fig 5D), which is comparable to that of the traditional AOSLO (70–100 μm) [5, 57]. By introducing defocus through the DM, we imaged the retina at different layers (Fig 5A–5C).

### Assess intra-frame distortion under different frame rates

**Fig 6:**
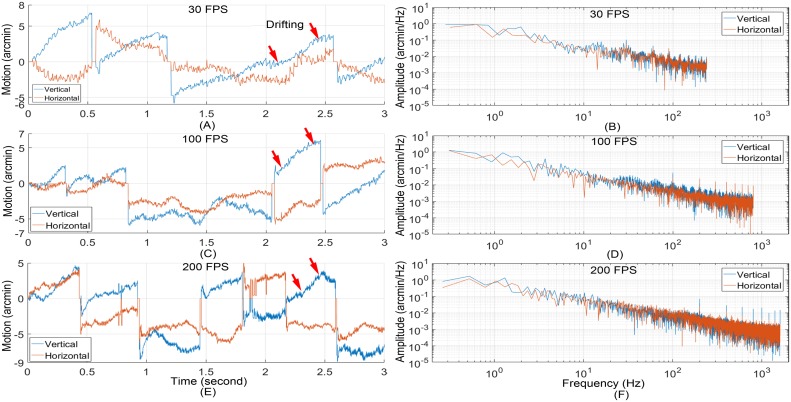
Retinal motion estimated with retinal images acquired at different frame rates. (A) Retinal motion trace calculated from images acquired at 30 FPS. (B) Power spectra of motion amplitude of (A). (C) Retinal motion trace calculated from images acquired at 100 FPS. (D) Power spectra of (C). Retinal motion trace calculated from images acquired at 200 FPS. (F) Power spectra of motion amplitude of (E). Red arrows indicate exemplary drifting motion periods.

In previous studies with AOSLO, we have learned that the eye movement during the drifting period between microsaccades causes the least distortion in retinal images among the three eye motion modes. This may be understood by the fact that this motion mode has relatively slow speed and small amplitudes. Thus, the retinal drifting period may be considered as an ideal time for acquiring image with minimum motion artifact. This condition is particularly in favor of low speed imaging. We thus compared the images taken with different frame rates during the smooth drifting periods between microsaccades (Fig 6).

Retinal images at the same location (1.5° eccentricity nasal) were acquired with frame rate of 30 FPS, 100 FPS, and 200 FPS. Eye motion was calculated following Vogal et al [35]. Fig 6 shows exemplary motion traces (Fig 6A, 6C and 6E) and the corresponding (amplitude) power spectra of the retinal motion estimated at each frame rate (Fig 6B, 6D and 6F). Microsaccades are the jerk like movement in the motion traces. The peak-to-valley (PV) values of vertical motion measured at 30 FPS, 100 FPS, and 200 FPS were 12.7 arcminutes, 12.0 arcminutes, and 13.4 arcminutes, respectively. The PV values of horizontal motion measured at 30 FPS, 100 FPS, and 200 FPS were 9.7 arcminutes, 9.6 arcminutes, and 11.3 arcminutes respectively. Because 16 stripes were used for motion analysis, the cut frequency for motion measurement at 30 FPS, 100 FPS, and 200 FPS were 240 Hz, 800 Hz, and 1600 Hz, respectively. The spikes in motion power spectra were caused by the flying-back motion of the scanning mirror.

**Fig 7:**
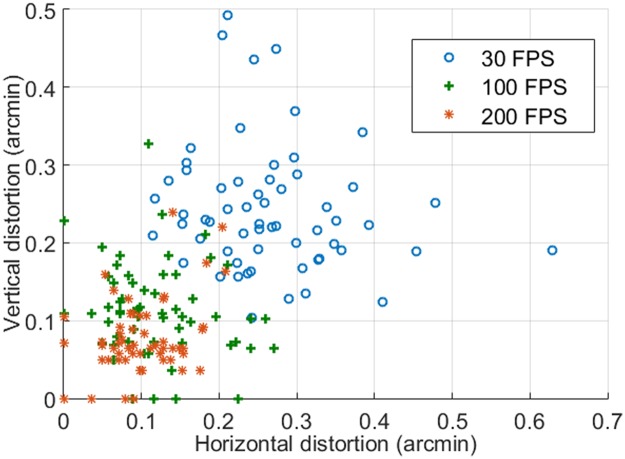
Image intra-frame distortions under different frame rates.

We selected 5 series of image sequences from each video recoded at different frames rates. Each period includes 12 successive frames acquired during a smooth drifting period (as illustrated in Fig 6A). The mean RMS of the eye movements of individual frames along X and Y direction are shown in Fig 7. The mean vertical distortion taken with 30 FPS, 100 FPS, and 200 FPS are 0.24 arcminute, 0.11 arcminute, and 0.08 arcminute, respectively; the corresponding mean horizontal distortions are 0.27 arcminute, 0.12 arcminute, and 0.11 arcminute, respectively.

**Fig 8:**
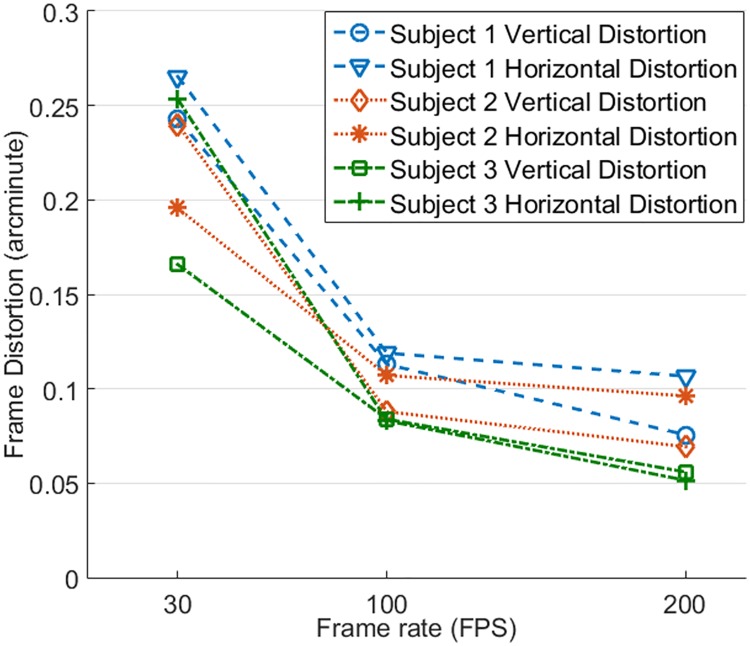
Intra-frame distortions of 3 subjects assessed with images acquired with different frame rates.

Intra-frame distortion has been assessed in 3 subjects in normal chorioretinal health (Fig 8). Image acquisition, motion analysis, and distortion estimation followed the same protocol. Again, image distortion was significantly reduced with increased frame rate. Compared with image acquired at 30 FPS, image distortions along X and Y direction for subject 1 were reduced by 55.2% and 53.5% at 100 FPS and by 59.8% and 68.9% at 200 FPS, for subject 2 were reduced by 45.3% and 63.3% at 100 FPS and by 50.8% and 71.1% at 200 FPS, and for subject 3 were reduced by 67.2% and 49.4% at 100 FPS and by 79.7% and 66.3% at 200 FPS. For all 3 subjects, vertical and horizontal distortions were less than 0.125 arcminute (~ 0.6 μm) when images were acquired with frames rate ≥ 100 FPS, approximately 1 pixel size in a typical AOSLO image, which was sufficient to ensure the structure of the smallest cells (foveal center cones, rods, whose inner segment diameter is about 2–3 μm) to be rendered with reduced distortion. The declination of distortion reduction from 100 FPS to 200 FPS becomes flatter than that from 30 FPS to 100 FPS.

## Discussion

In this study, we demonstrated the feasibility to take confocal retinal images in the living human eye with a speed that can minimize the retinal motion artifact. The device acquired a frame within a time period of 5 ms (for an image size similar to that of classical AOSLO), which is close to the typical single frame acquisition time (4 ms) of an AO-fundus camera [58–60].

High resolution retinal imaging with minimal motion artifact is critical for sensitive and precise assessment of retinal structure and its change over time. High speed image acquisition is one of the solutions for overcoming motion artifact. AO-fundus cameras operate with a flood-illumination mode and can acquire the retinal image by a ‘snap-shot’ [58–60], during which the retinal motion causes little artifact thus the image can be considered as distortionless. However, the contrast of the images taken by an AO-fundus camera tends to be compromised by scattering from different retinal layers. Adaptive optics assisted confocal retinal imaging (as represented by AOSLO) possesses the ability to reject the out-of-focus scattering but is plagued by retinal motion artifact. Our development has bestowed high speed on AO confocal retinal imaging.

AO line scan retina imaging concept has been reported by Mujat et al [42]. Our system has a more advanced design and has yielded improved performance. Compared with the pioneer work of Mujat et al, in addition to the significantly increased image acquisition speed (200 FPS vs. 15 FPS), our system operates with a field of view (1.2° vs. 5.5°) within the isoplanatic zone of the ocular wave aberration thereby ensuring a uniform correction for wave aberration across the whole imaging field. Furthermore, our system possesses a higher digitization rate (0.67 μm/pixel vs. 1.56 μm/pixel) that ensures fine retinal structure such as rod photoreceptors (whose inner segments’ diameter is of 2~3 μm) to be rendered with sufficient pixels. With robust AO compensation for ocular wave aberration, cones in the fovea were resolved as near as to 0.2° eccentricity and rods were imaged near the foveal (Fig 4), whereas Mujat’s system could resolve cones at the eccentricity > 1° only and did not image rods. The high speed imaging system has an ability of depth discrimination comparable to that of traditional AOSLO and sufficient to reveal retinal structure of different layers (Fig 5).

An important technical point worth noting is that our high speed imaging was achieved over a field of view 1.2° × 1.2° in the eye with a digitization of 512 × 512 pixels/frame, rendering a reasonable size of the retina with sufficient optical resolution and digitization. The frame rate can be as high as 1600 FPS with a digitization of 512 × 64 pixels/frame (corresponding to a field size of 1.2° × 0.15°). AO-fundus cameras with a similar speed have been reported previously, e.g., Beggood et al achieved a frame rate up to 1 KHz [61] with images acquired within a field of view (1.2° × 0.14°). While this setting satisfies certain studies, it is impractical for applications requiring survey of a large retinal area.

Evidently, with increased frame rate, the intra-frame image distortion was reduced. Notably, the minimum distortion in 30 FPS images was greater than 0.1 arcminute, whereas in 100 FPS and 200 FPS video, the minimum distortion could be as low as 0, indicating the possibility to achieve distortionless imaging with high frame rate. Although the stripe-based image registration method [35] has provided a robust strategy for correction of distortions and averaging successive frames, the accuracy of this method relies on the reference frame, which must be carefully selected to be with minimum distortion. A reference image plays a critical role for high precision image registration. High speed imaging may facilitate better and easier selection or generating a reference frame with minimal intra-frame distortion.

The image acquisition speed (200 FPS) achieved in this study represents a continuum effort on making practical AO confocal imaging technology. Indeed, low speed retinal imaging with sophisticated post or real time processing have provided decent image quality [29, 35] and enabled important studies, but in general high speed imaging is desirable for both basic science and clinical research. The benefit of high speed imaging can also be demonstrated by improved image acquisition efficiency in obtaining large retina image montage. Because high resolution images can be acquired within a small field (typically ~1°) only, a large field image must be montaged from individual images taken at different retinal locations. At each place, successive frames must be recorded (usually about 10–20 seconds) and added to reduce noise and generate a stable image. With a frame rate of 30 FPS and digitization of 512 × 512 pixels/frame, an imaging session to generate a montage of the macula often takes more than an hour. This is a rather inefficient procedure and imposes a big obstacle for clinical applications involving elderly adults and young children, because their ability to maintain stable fixation in such an exhausting time may be limited. Moreover, retinal disease such as retinitis pigmentosa, achromatopsia, congenital stationary night blindness and Stargardt disease are often associated with either nystagmus or unsteady fixation due to central vision loss. Thus, high speed imaging taken with ‘a snap-shot’ can reduce the overall imaging time.

We have identified important limitations that are addressable in future study. First, the resolution and contrast of high speed line scanning imaging system are intrinsically compromised along the line direction of the camera. This problem may be mitigated by use of parallel point light source or structure illumination [62, 63]. Second, to ensure sufficient digitization for optical resolution, the camera collects only a small portion (0.28 of the Airy disc diameter) of imaging light along the image line, because the camera has two lines of pixels only. While this configuration ensures highly confocal imaging, the loss of imaging light is significant. This problem may be solved by use of high speed camera with multiple lines of pixels available in the future, or by advanced optical design with non-symmetrical magnification that can compress imaging light onto the line image chip of the camera while maintaining optical magnification along the line direction. Third, image brightness is relatively low compared to classical AOSLO that uses photomultiplier tubes or avalanche photodiodes which have a much higher gain. Fourth, to achieve a good SNR when using a 200 FPS imaging rate, this imaging system required that the power of the imaging light at the eye (880 μW) be approximately 1.8 to 4 times higher than powers commonly reported in other AOSLO systems for similar imaging wavelengths (230–500 μw) [8, 47, 64, 65]. However, we note that our imaging light is focused to a line on the retina, thereby spreading the power over a large area (as opposed to most AOSLO systems in which the power is focused in a more concentrated manner into a small, near-diffraction-limited scanning point), and was still below the ANSI standard. With high quantum efficiency and high gain photodetector array available in the future, we expect that these questions may be solved. Furthermore, methods reducing torsional distortion of the image remains to be investigated.

## Conclusions

We have demonstrated a high speed AO line scan confocal retinal imaging system. High-speed image acquisition (up to 200 FPS) significantly improved data collection efficiency and effectively eliminates image distortion caused by eye movement. This instrument may facilitate studies involving subjects with nystagmus or unsteady fixation due to central vision loss.
